# Ctt1 catalase activity potentiates antifungal azoles in the emerging opportunistic pathogen *Saccharomyces cerevisiae*

**DOI:** 10.1038/s41598-019-45070-w

**Published:** 2019-06-24

**Authors:** Dorival Martins, Dao Nguyen, Ann M. English

**Affiliations:** 1 Department of Chemistry and Biochemistry, Concordia University, 7141 Sherbrooke Street West, Montreal, Quebec H4B 1R6 Canada; 2 McGill University Health Centre Research Institute and Meakins-Christie Laboratories, 1001 Boulevard Decarie West, Montreal, Quebec H4A 3J1 Canada

**Keywords:** Drug development, Antimicrobial responses, Fungal infection, Stress signalling, Metalloproteins

## Abstract

Fungi respond to antifungal drugs by increasing their antioxidant stress response. How this impacts antifungal efficacy remains controversial and not well understood. Here we examine the role of catalase activity in the resistance of *Saccharomyces cerevisiae* to the common antifungals, fluconazole and miconazole, for which we report minimum inhibitory concentrations (MICs) of 104 and 19 μM, respectively. At sub-MIC concentrations, fluconazole and miconazole stimulate catalase activity 2-3-fold but, unexpectedly, deletion of cytosolic catalase (*ctt1*) makes cells more resistant to these azoles and to clotrimazole, itraconazole and posaconazole. On the other hand, upregulating Ctt1 activity by preconditioning with 0.2 mM H_2_O_2_ potentiates miconazole 32-fold and fluconazole 4-fold. Since H_2_O_2_ preconditioning does not alter the resistance of *ctt1*Δ cells, which possess negligible catalase activity, we link azole potentiation with Ctt1 upregulation. In contrast, *sod2Δ* cells deleted for mitochondrial superoxide dismutase are 4–8-fold *more* azole sensitive than wild-type cells, revealing that Sod2 activity protects cells against azole toxicity. In fact, the *ctt1*Δ mutant has double the Sod2 activity of wild-type cells so *ctt1* deletion increases azole resistance in part by Sod2 upregulation. Notably, deletion of peroxisomal/mitochondrial *cta1* or cytosolic *sod1* does not alter fluconazole or miconazole potency.

## Introduction

Antimicrobial challenge appears to induce the rewiring of microbial metabolic networks and stress-response pathways regardless of the primary drug-target interaction^1,2^. Drug lethality increases when major stress responses are disrupted^3–11^ whereas resistance increases in fungi^12–14^ and bacteria^3,4,7,9,15–17^ when antioxidant defenses are boosted. Susceptibility to antimicrobial killing decreases when cells are treated with antioxidants^1,2,13^. Moreover, deletion of respiratory enzymes or inhibition of cellular respiration, a major source of reactive oxygen species (ROS)^1,2,18^, decreases antimicrobial lethality. Combined, these observations are consistent with the belief that cidal antibiotics^1,19^ and antifungals^2,20,21^ increase ROS levels. Hence, understanding the roles of ROS-metabolizing enzymes in antimicrobial efficacy is of critical importance in treating infection.

Several studies have examined the contribution of key ROS-metabolizing enzymes such as catalases and superoxide dismutases (Sods) to bacterial survival following challenge with antibiotics. For example, the *ΔrelA ΔspoT* mutant of *Pseudomonas aeruginosa*, which is deficient in the (p)ppGpp alarmone, exhibits depressed catalase and superoxide dismutase (Sod) activities and is hypersensitive to antibiotics^3,4,22^. This can be reversed by overexpression in the mutant of KatA, the dominant *P. aeruginosa* catalase^4^, or by restoration of Sod activity^22^. Many other bacteria become more susceptible to antibiotics on Sod deletion, including *Enterococcus faecalis*^9,23,24^, *Campylobacter jejuni*^8^, *Acinetobacter baumanii*^7^, *Staphylococcus aureus*^24^ and *E. coli* in stationary-phase^25^ but maybe not in exponentially growing cultures^15^. Deletion of the catalase-peroxidase *katG* or the alkyl hydroperoxide reductase *ahpC* also potentiates some antibiotics in *E. coli*^15^.

**Figure 1:**
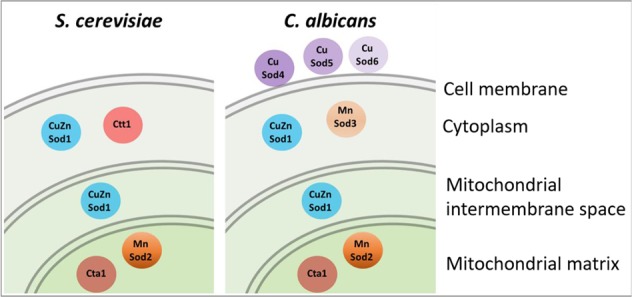
Subcellular localization of catalases and superoxide dismutases (SODs) in *S. cerevisiae* and *C. albicans*. Cytosolic Ctt1 is found in the cytoplasm of *S. cerevisiae*, whereas Cta1 is co-targeted to peroxisomes and mitochondria in respiring *S. cerevisiae*^48^ and inferred to be associated with these two compartments in *C. albicans*. CuZnSod1 is localized in the cytoplasm and the mitochondrial intermembrane space of both yeasts, while MnSod2 is present in the mitochondrial matrix. *C. albicans* possesses an extra MnSod3 in the cytosol and cell-membrane-associated CuSod4-6, which are absent in *S. cerevisiae*. Note that *C. glabrata* possess only Cta1^5^, CuZnSod1 and MnSod2^74^.

Antioxidant enzymes also are associated with antifungal potency. For example, sirtuin Hst1 deletion increases catalase activity and lowers multidrug sensitivity in *Candida glabrata*^5^. Deletion of membrane-associated CuSod4 and CuSod5^26^ (Fig. 1) or inhibition of Cu-dependent Sod activity in *Candida albicans* increases the anitbiofilm activity of miconazole^13^ and amphotericin B^27^. Fluconazole induces a number of genes responsive to oxidative- and nitrosative-stress in *C. albicans*^28^ and both fluconazole- and amphotericin B-resistant *C. albicans* and *Candida dubliniensis* exhibit increased catalase and Sod activities^12^.

Previously, it was shown that exposure to a fungistatic dose of miconazole induces catalase activity in both *C. albicans* and *Saccharomyces cerevisiae*^14^. Notably, the catalase and superoxide dismutase isoforms present in these yeasts differ considerably (Fig. 1). *C. albicans* possesses a single peroxisomal/mitochondrial catalase (Cta1)^29^ together with six Sods^13^ while *S. cerevisiae* produces cytosolic Ctt1 in addition to Cta1 but just two Sods, cytosolic CuZnSod1, which also localizes to the mitochondrial intermembrane space^30,31^, and mitochondrial MnSod2^32^ (for clarity, we indicate Sod metal dependence throughout the text). Thus, a comparison of how deletion of specific antioxidant enzymes alters antifungal potency in these well-characterized yeasts provides an excellent opportunity to gain new insights into pathogen survival strategies and the evolution of antifungal resilience.

In this work, we focus on the role of catalase activity in the response of *S. cerevisiae* (strain BY4741; Table 1) to challenge with common antifungal azoles. The primary target of these drugs is ergosterol biosynthesis^33^, a sterol found in the cell membrane of fungi. Specifically, we report on the azole resistance of single *ctt1* and *cta1* knockouts (Table 1) as well as on wild-type cells preconditioned with a low dose of H_2_O_2_ to stimulate catalase activity^34,35^. Furthermore, since MnSod2 is induced by the H_2_O_2_ stress response^17,36,37^, we also monitored the Sod activity and azole sensitivity of *sod1* and *sod2* mutants (Table 1) with and without catalase inhibition. Combined, our unprecedented results shed new light on antioxidant defense and azole resistance in *S. cerevisiae*, which itself is an emerging opportunistic pathogen^38–41^.

**Table 1:** *S. cerevisiae* strains used in this study.

Strain	Description	Source
wild-type BY4741	*MATa his3Δ1 leu2Δ0 met15Δ0 ura3Δ0*	EUROSCARF
*cta1*∆ strain	BY4741 cells with *cta1::KAN4MX*	See ref.^34^
*ctt1*∆ strain	BY4741 cells with *ctt1::KAN4MX*	See ref.^34^
*sod1*∆ strain	BY4741 cells with *sod1::KAN4MX*	C. Brett, Concordia U
*sod2*∆ strain	BY4741 cells with *sod2::KAN4MX*	C. Brett, Concordia U

## Results

### MICs of azoles for *S. cerevisiae* and their classification as fungicidal vs. fungistatic

**Figure 2:**
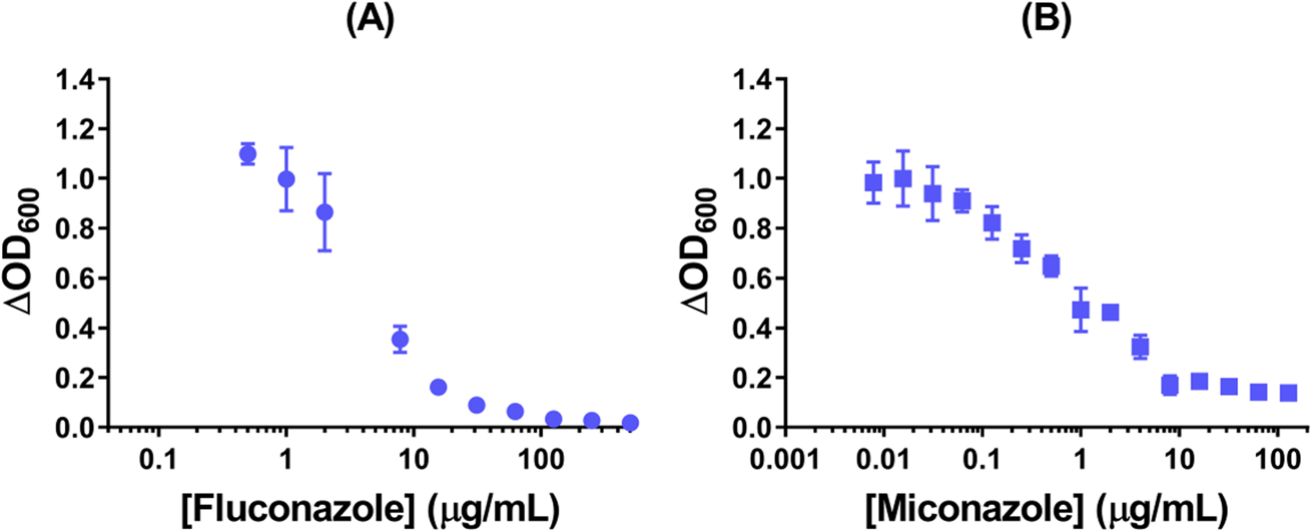
Minimum inhibitory concentration (MIC) of fluconazole and miconazole for wild-type *S. cerevisiae* cells. Wild-type cells grown to OD_600_ 0.50 (12 h) in YPD at a medium-to-flask ratio of 1:5 at 30 °C/225 rpm were diluted to OD_600_ 0.15 (10^6^ cfu/mL) before challenge with increasing azole concentration in a 96-well plate. OD_600_ values were read in the plate reader at time t = 0, and cells were incubated at 30 °C without stirring. OD_600_ values were read again in the plate reader at t = 24 h, and ΔOD_600_ (24–0 h) values were plotted vs. (**A**) Fluconazole and (**B**) miconazole concentration to give MIC of 32 and 8 μg/mL, respectively. The results represent the avg ± SEM of six independent replicates (*n* = 6).

Starting at an initial cell density of 10^6^ cfu/ml and based on cell growth at different drug concentrations (Fig. 2), we determined the minimum inhibitory concentration (MIC µg/mL; µM) for our *S. cerevisiae* strain (BY4741) of six medically relevant azoles: itraconazole (32; 45), fluconazole (32; 105), posaconazole (32; 46), voriconazole (>256; >730), miconazole (8; 19) and clotrimazole (4; 12) (Table S1). The structures of the azoles, shown as a footnote to Table S1, reveal that the drugs examined can be classified as triazoles (itraconazole, fluconazole, posaconazole and voriconazole) and imidazoles (miconazole and clotrimazole). The imidazoles are more potent antifungals than the triazoles and, in fact, cells are refractory to voriconazole (Table S1). An azole is classified as fungicidal if 1xMIC or 2xMIC promotes a ≥10^3^-fold reduction in the viable cfu/mL and Table S2 shows that the imidazoles are fungicidal under the present experimental conditions, whereas the triazoles are fungistatic with the exception of voriconazole.

Cultures of *C. albicans* (strain SC5314) at the same initial cell density (10^6^ cfu/mL) exhibit MICs of >1 mM for fluconazole^42^ and 60 µM for miconazole^43^. Thus, under our culture conditions, *S. cerevisiae* strain BY4741 is more sensitive to the azoles than *C. albicans* strain SC5314^42,43^. We note that our initial cell density is higher than that used in standardized methods of antifungal susceptibility testing recommended by the Clinical and Laboratory Standards Institute (10^3^ cfu/mL)^44^ to provide sufficient cells for the biochemical analyses. At high cell density, the tolerance for the azoles is likely higher than under standard testing conditions^42^. However, relative MICs are of interest here and in the further studies described here, fluconazole and miconazole represent the fungistatic triazoles and fungicidal imidazoles, respectively.

### Fluconazole and miconazole at sub-MIC increase catalase activity and H_2_O_2_ levels in *S. cerevisiae*

**Figure 3:**
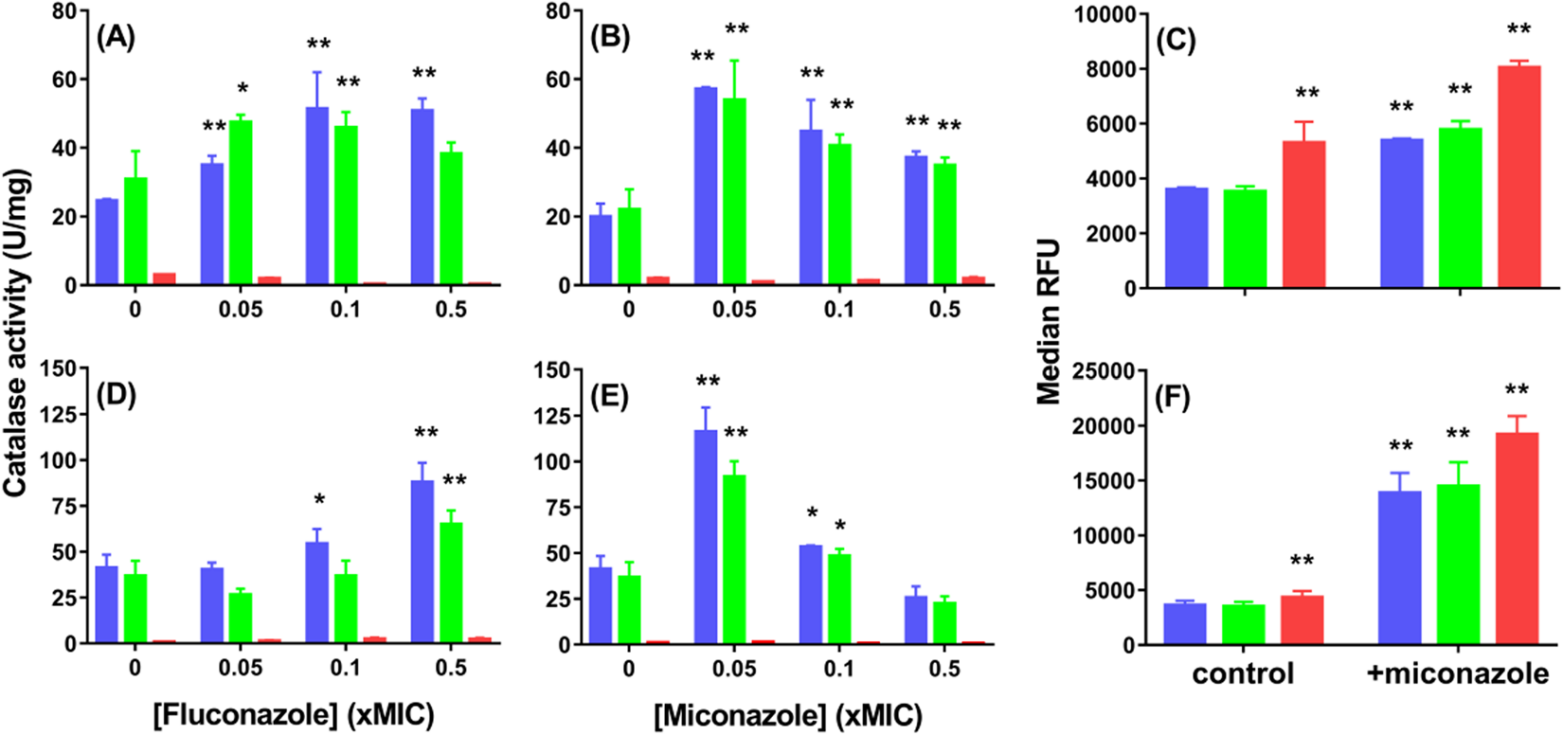
Azoles and H_2_O_2_ stimulate catalase activity in wild-type and cta1Δ cells but not in ctt1Δ cells. Total catalase activity was measured without (**A**,**B**) and with (**D**,**E**) H_2_O_2_ preconditioning of wild-type , *cta1*Δ  and *ctt1*Δ cells  at 24 h after challenge of 3-mL cultures at 10^6^ cfu/mL with (**A**,**D**) fluconazole and (**B**,**E**) miconazole at concentrations below their minimum inhibitory concentrations (MICs; see Table 2). Relative intracellular H_2_O_2_ levels measured by flow cytometry at (**C**) 8 h and (**F**) 24 h after DHR-stained wild-type , *cta1*Δ  and *ctt1*Δ cells  were exposed to 0.05xMIC miconazole or ethanol only (control). Experimental conditions: Cells at an initial OD_600_ of 0.15 were grown in YPD at a medium-to-flask ratio of 1:5 at 30 °C/225 rpm. Catalase activity was assayed (see *Materials and Methods*) at 24 h after 3-mL cultures were challenged with azole in 12 μL of ethanol. For preconditioning, cultures were grown to OD_600_ 0.50 (12 h), 0.2 mM H_2_O_2_ was added to the medium, cells were diluted 30 min later to OD_600_ 0.15 (10^6^ cfu/mL) and challenged with azole in 3-mL cultures. To determine relative H_2_O_2_ levels, cells grown in 3-mL cultures at initial OD_600_ of 0.15 were was stained with 5 µM DHR in 1-mL aliquots at 30 °C, pelleted after 120 min, diluted to 10^6^ cells/mL in PBS, fixed with 2% formalin (v/v) and analyzed by flow cytometry (see *Materials and Methods*). Relative fluorescence units (RFU; ex/em 490/520 nm) of individual cells were measured and the median RFU of 10,000 cells estimates a sample’s relative H_2_O_2_ level. All results represent the avg ± SEM of six independent experiments (*n* = 6). Statistical analyses performed using Student’s t-test compare each sample with the wild-type untreated control. *p < 0.05 and **p < 0.01.

Both azoles increase catalase activity in wild-type *S. cerevisiae* (Fig. 3A,B)^14^. However, a 10-fold lower concentration of miconazole (~1 μM; 0.40 μg/mL) vs. fluconazole (~10 μM; 3.2 μg/mL) doubles the catalase activity of wild-type cells as measured at 24 h after azole addition (Fig. 3A,B). Notably, catalase activity declines when cells are treated with increasing miconazole concentrations (Fig. 3B), which concurs with the report that fungicidal doses deplete catalase activity in *S. cerevisiae* and *C. albicans*^14^.

Fungicidal miconazole also induces more ROS formation than fungistatic fluconazole in wild-type *S. cerevisiae*^2^, *C. albicans*^2^ and *C. glabrata*^45^. To examine the rise in intracellular H_2_O_2_ induced by the fungicide, we stained cells with the profluorescent dye DHR, which is preferentially oxidized by H_2_O_2_ in the presence of cellular catalysts^46^. At 8 and 24 h after challenge of wild-type cells with 0.4 μg/mL miconazole, we observe a 1.5- and 3-fold increase in probe fluorescence, respectively, compared to untreated control cells (Fig. 3C,F). Hence, a high level of H_2_O_2_ accumulates over time in miconazole-treated wild-type cells, affirming that the fungicidal azole increases ROS in yeast^2,20^.

### *Ctt1* catalase activity weakly combats miconazole-induced H_2_O_2_ accumulation in *S. cerevisiae* cells

Miconazole induces a rise in intracellular H_2_O_2_ (Fig. 3C,F) despite also inducing catalase activity in wild-type cells. This led us to examine catalase activity and H_2_O_2_ levels in the *cta1*Δ and *ctt1*Δ strains, which lack peroxisomal/mitochondrial and cytosolic catalase, respectively (Fig. 1). Catalase activity (Fig. 3A,B) and H_2_O_2_ levels (Fig. 3C) are the same in wild-type and *cta1*Δ cells, which reflects the strong repression of Cta1 by glucose in the medium^34,47,48^. In contrast, Ctt1 is not repressed by glucose^34,49,50^ and confers most of the catalase activity in cells grown in YPD since *ctt1*Δ cells are virtually devoid of catalase activity (Fig. 3A,B). Moreover, the azoles fail to induce catalase activity in the *ctt1*Δ strain (Fig. 3A,B) although ~4 times more H_2_O_2_ accumulates in miconazole-treated vs. control over 24 h (Fig. 3F). It also is remarkable that the H_2_O_2_ levels in wild-type and *cta1*Δ cells are ~75% those of *ctt1*Δ cells with negligible catalase activity (Fig. 3C,F). Thus, Ctt1 appears to be an ineffective scavenger of miconazole-induced H_2_O_2_.

### Deletion of *ctt1* or inhibition of catalase activity increases azole resistance in *S. cerevisiae*

Peroxide-metabolizing enzymes have been associated with protection against cidal antimicrobials^4,5,8,14,15,45^. However, our observation that Ctt1 does not inhibit miconazole-induced H_2_O_2_ accumulation (Fig. 3C,F) led us to ask whether Ctt1 actually protects cells against azole toxicity. As shown in Table 2 and Fig. S1, *ctt1*Δ cells display 4- and 8-fold *higher* MICs for fluconazole and miconazole, respectively, than the two strains with Ctt1 activity (Tables 2, S1). Given this surprising observation, we additionally determined the fold-change in MIC when *ctt1* was deleted for the four other azoles. Both wild-type and *ctt1*Δ cells are refractory to voriconazole (Table S1) but the *ctt1*Δ strain is 8-fold less sensitive to posaconazole and 2-fold less sensitive to clotrimazole and itraconazole than wild-type cells (Table S1). Thus, Ctt1 appears to *potentiate* both fungistatic and fungicidal azoles in *S. cerevisiae*.

**Table 2:** Fluconazole and miconazole MICs for wild-type and mutant *S. cerevisiae* cells ± H_2_O_2_ preconditioning and ± aminotriazole^a^.

MIC (µg/mL)^b^					
Strain^c^	Fluconazole		Miconazole		
	saline^d^	+H_2_O_2_^d^	saline^d^	+H_2_O_2_^d^	+ATZ^f^
wild-type	32 (2A)	8 (S2A)	8 (2B)	0.25 (S2B)	32
*cta1*Δ	32 (S1A)	8 (S3A)	8 (S1B)	0.5 (S3B)	ND^e^
*ctt1*Δ	128 (S1C)	128 (S3C)	64 (S1D)	32 (S3D)	32
*sod1*Δ	32 (S4A)	ND^e^	8 (S4B)	ND^e^	ND^e^
*sod2*Δ	8 (S4C)	ND^e^	1 (S4D)	ND^e^	4

^a^Minimum inhibitory concentrations (MICs) for cultures diluted to an initial cell density of 10^6^ cfu/mL before challenge. Growth conditions are given in the legend to Fig. 2. Cultures were preconditioned with saline (0.85% wt/v aqueous NaCl) or 0.2 mM H_2_O_2_ for 30 min under the same conditions.

^b^Note that 1.0 μg/mL corresponds to 3.3 μM fluconazole (MW 306 Da) and 2.4 μM miconazole (MW 416 Da).

^c^The strains are described in Table 1.

^d^MICs were determined from plots of OD_600_ vs. [azole] shown in the figures listed in the parentheses.

^e^ND = not determined.

^f^Aminotriazole (ATZ) was present at 25 mM during the incubation with miconazole.

Aminotriazole is a well-documented inhibitor of catalase activity in *S. cerevisiae*^51^. Thus, to directly probe the effect of inhibition of catalase activity on miconazole resistance we added aminotriazole to the cells. This compound did not inhibit the growth of any strain at concentrations as high as 100 mM (data not shown) but treatment with 25 mM lowers catalase activity to undetectable levels in wild-type cells (Fig. S5). In the presence of 25 mM aminotriazole, both wild-type and *ctt1*Δ cells have the same miconazole MIC (32 μg/mL; Table 2), which links miconazole potentiation in wild-type cells with Ctt1 catalase activity.

### H_2_O_2_ preconditioning stimulates Ctt1 catalase activity and lowers the fluconazole and miconazole resistance of *S. cerevisiae* cells

Prompted by the link between miconazole potentiation and catalase activity, we questioned whether stimulating this activity before azole addition would further sensitize cells to the drug. As we previously reported^34^, preconditioning wild-type or *cta1*Δ cells with a low dose of H_2_O_2_ (e.g., 0.2 mM) in YPD medium doubles their Ctt1 activity (Fig. 3D,E vs. 3A,B; no azole). The combination of H_2_O_2_ preconditioning and azole challenge (0.5xMIC fluconazole or 0.05xMIC miconazole) increases Ctt1 activity by 4–6-fold above basal levels in wild-type and *cta1*Δ cells (Fig. 3D,E vs. A,B) and increases their azole sensitivity 4–32-fold (Table 2). In contrast, H_2_O_2_ preconditioning has little or no effect on the catalase activity (which remains barely detectable; Fig. 3) or azole sensitivity of the *ctt1*Δ strain (Table 2), affirming that azole potentiation is linked to increased Ctt1 activity and is not augmented by H_2_O_2_ exposure. Moreover, H_2_O_2_ preconditioning potentiates miconazole significantly more than fluconazole (Table 2) presumably because the fungicide is the more potent stimulator of Ctt1 activity (Fig. 3B,E vs. 3A,D).

### Deletion of *ctt1* elevates MnSod2 activity in early log phase and increases miconazole resistance

**Figure 4:**
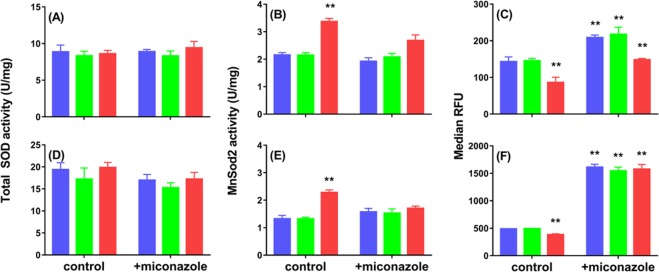
MnSod2 activity is higher and O_2_^•−^ levels lower in ctt1Δ cells vs. wild-type and cta1Δ cells. (**A**,**D**) Total Sod activity (CuZnSod1 plus MnSod2), (**B**,**E**) MnSod2 activity, and (**C**,**F**) relative intracellular O_2_^•−^ levels measured by flow cytometry with DHE staining at (**C**) 8 h and (**F**) 24 h after wild-type , *cta1*Δ  and *ctt1*Δ cells  (see Fig. 2) at 8 h (top panels) and 24 h (bottom panels) after the addition of 0.05xMIC (0.4 μg/mL) miconazole (+miconazole) or ethanol vehicle (control) to the cultures. The Sod activity assay is described under *Materials and methods*. KCN (5 mM) was added to selectively inhibit CuZnSod2 activity in (**B**,**E**). To determine relative O_2_^•−^ levels, cells were grown in 3 mL cultures at an initial OD_600_ of 0.15, and 1 mL of culture was stained with 5 µM DHE at 30 °C, pelleted after 60 min, diluted to 10^6^ cells/mL in PBS, fixed with 2% formalin (v/v) and analyzed using flow cytometry (see *Materials and Methods*). Relative fluorescence units (RFU; ex/em 490/580 nm) of individual cells were measured and the median RFU of 10,000 cells estimates a sample’s relative O_2_^•−^ levels. Results represent the avg ± SEM of six independent experiments (*n* = 6). Statistical analyses performed using Student’s t-test compare each sample with the wild-type untreated control. **p < 0.01.

Although fungicide-dependent ROS production reportedly leads to fungal cell death^2,13,20^, we find no link here between elevated H_2_O_2_ levels and miconazole sensitivity. In fact, *ctt1*Δ cells, which are the most miconazole resistant (Table 2), accumulate more H_2_O_2_ on challenge with this azole (Fig. 3C,F). However, the miconazole resistance of *C. albicans* biofilms is dependent on the ROS-detoxifying activity of Sods^22^, and we^37^ and others^36^ have shown previously that suppressing or deleting catalase activity in *S. cerevisiae* upregulates mitochondrial MnSod2. Thus, we hypothesized that increased MnSod2 activity contributes to the enhanced azole resistance of *ctt1*Δ cells (Table 2). There are two Sod isoforms in *S. cerevisiae* (Fig. 1), and we find that the three strains exhibit similar *total* Sod activity, which doubles between 8 and 24 h but does not increase upon miconazole challenge (Fig. 4A,D). Since MnSod2 accounts for only 10–20% of the total Sod activity in cells growing on glucose^52^, to unmask any variation in this activity, we selectively inhibited CuZnSod1 with KCN^53^. This revealed 1.7-fold higher MnSod2 activity in untreated *ctt1*Δ cells vs. wild-type or *cta1*Δ cells (Fig. 4B,E).

We next compared the relative levels of O_2_^•−^ in the three strains. Staining cells with the profluorescent dye, DHE, which is preferentially oxidized by O_2_^•−^ ^54^, we uncovered 2-fold less O_2_^•−^ in the *ctt1*Δ strain relative to wild-type or *cta1*Δ cells (Fig. 4C). O_2_^•−^ levels were a factor of ~1.3 higher in the cultures challenged with miconazole but remained significantly lower in *ctt1*Δ cells (Fig. 4C). The O_2_^•−^ levels tripled between 8 and 24 h such that the 24-h miconazole-challenged cells contained > 10-fold more O_2_^•−^ than the untreated 8-h cells (Fig. 4F vs. 4C). Also, the 24-h cultures have comparable O_2_^•−^ levels and MnSod2 activity (Fig. 4E,F) so azole resistance must be associated with the O_2_^•–^detoxifying activity of MnSod2 during exponential growth. Thus, we conclude that *ctt1*Δ cells are more azole resistant (Table 2) because they possess the higher MnSod2 activity in early log phase (Fig. 4B,C).

We additionally examined if the Sod mimetics, TEMPO^•^ or mito-TEMPO^•^, protect wild-type *S. cerevisiae* against miconazole toxicity. These radicals are well-established O_2_^•−^ scavengers and mito-TEMPO^•^ is targeted to mitochondria^55^ but not TEMPO^•^ ^55,56^. Addition of 1 mM TEMPO^•^ did not change the MIC of miconazole (Table 3) and 1 mM mito-TEMPO^•^ afforded only modest protection, doubling the MIC of miconazole in four of the six independent cultures examined (Table 3). It is possible that O_2_^•−^ scavenging by mito-TEMPO^•^ is offset by the miconazole-induced increase in respiration in wild-type cells (Fig. S6). Increased respiration is not detected in the *ctt1*Δ mutant (Fig. S6) so its elevated MnSod2 activity may provide better protection against miconazole-dependent O_2_^•−^ production than MnSod2 plus mito-TEMPO^•^ in wild-type cells. Also, the efficacy of mito-TEMPO^•^ may be lowered by its reaction with mitochondrial reductases^55^.

**Table 3:** Miconazole MIC for fermenting wild-type *S. cerevisiae* cells ± O_2_^•−^ scavengers^a^.

MIC (µg/mL)^a^		
Miconazole	Miconazole + 1 mM TEMPO^•^	Miconazole + 1 mM mito-TEMPO^•^
8 (6)^b^	8 (6)^b^	16 (4)^b^ 8 (2)^b^

^a^See Footnotes *a-c* to Table 2.

^b^The number of independent observations of a given MIC is in brackets in red font.

### *S. cerevisiae* cells deleted for *sod2*Δ exhibit decreased fluconazole and miconazole resistance

To further explore the importance of Sod activity in azole resistance, we measured the fluconazole and miconazole MICs for *sod1*Δ and *sod2*Δ cells. MICs are the same for *sod1*Δ and wild-type cells, revealing that CuZnSod1 deletion does not impact miconazole resistance (Table 2), which is consistent with 1 mM TEMPO^•^ having no protective effect (Table 3). However, the *sod2*Δ strain possesses fluconazole and miconazole MICs that are 4- and 8-fold lower, respectively (Table 2). These results confirm that MnSod2 activity protects cells from azole toxicity and upregulation of MnSod2 activity in the *ctt1*Δ strain (Fig. 4B,E) increases its azole resistance (Table 2).

### Inhibiting catalase activity in the *sod2*Δ strain enhances miconazole resistance less than in wild-type cells

If Ctt1 activity potentiates the azoles by suppressing MnSod2, then inhibiting catalase activity in the *sod2*Δ strain should not enhance resistance. Treatment of *sod2*Δ cells with 25 mM aminotriazole resulted in undetectable catalase activity as seen for wild-type cells (Fig. S5). The MIC for miconazole increased from 1 to 4 µg/mL vs. the increase to 32 µg/mL seen on aminotriazole treatment of wild-type cells (Table 2). Hence, Ctt1 activity potentiates miconazole in large part by depressing MnSod2 activity or in other words, the O_2_^•−^ detoxifying activity of MnSod2 combats azole toxicity and its deletion or suppression by Ctt1 activity lowers azole resistance in *S. cerevisiae*.

## Discussion

### Cytosolic Ctt1 catalase activity, not elevated intracellular H_2_O_2_, potentiates azole toxicity

De Nollin *et al*. found that fungistatic doses of miconazole stimulate catalase activity in *S. cerevisiae*^14^ and proposed that this rescues cells from H_2_O_2_ intoxication. We report here that sub-MIC concentrations of miconazole induce Ctt1 catalase activity up to 3-fold in our wild-type *S. cerevisiae* strain (BY4741) (Fig. 3) but this does prevent cells from accumulating ~4-fold more H_2_O_2_ over 24 h than untreated cells (Fig. 3F). Furthermore, *ctt1*Δ cells with negligible catalase activity, accumulate more miconazole-induced H_2_O_2_ than wild-type or *cta1*Δ cells (Fig. 3) but *are* more resistant to the azole (Table 2). Therefore, contrary to expectation^14^, our results reveal that azole-induced H_2_O_2_ production does not alter cell viability. In fact, cytosolic Ctt1 ineffectively combats H_2_O_2_ accumulation in wild-type and *cta1*Δ cells, and the increased azole sensitivity of H_2_O_2_-preconditioned wild-type cells results from Ctt1 upregulation and not exposure to exogenous H_2_O_2_. In sum, the azoles potentiate their own toxicity by induction of Ctt1 and not H_2_O_2_.

### Cytosolic Ctt1 activity potentiates the azoles partly by depressing MnSod2 activity

Since *ctt1*Δ cells with the highest MnSod2 activity of the strains examined here (Fig. 4) are 4–8-fold more azole resistant than wild-type cells, we conclude that depression of MnSod2 activity on Ctt1 stimulation potentiates the azoles. MnSod2 is not essential for fermenting *S. cerevisiae*^57^ but *sod2*Δ cells exhibit 4–8-fold lower azole resistance than wild-type cells (Table 2). However, we note that H_2_O_2_ preconditioned wild-type cells are 4-fold more miconazole sensitive than *sod2*Δ cells (Table 2). Thus, strong Ctt1 induction may potentiate miconazole by additional mechanisms. For example, miconazole may bind to the heme of Ctt1 as reported for CYP51^58^, the 14α-demethylase in the ergosterol biosynthetic pathway^33^. This could promote heme-catalyzed azole autoxidation with the formation of reactive, cytotoxic species via mechanisms analogous to those we reported for hydrazides^59^.

### Elevated MnSod2 activity in early log phase increases azole resistance

The *ctt1*Δ cells from 8-h cultures possess higher MnSod2 activity and less O_2_^•−^ than wild-type and *cta1*Δ cells (Fig. 4). Respiration is a major source of O_2_^•−^, and induction of respiration by miconazole reportedly increases its toxicity in *S. cerevisiae* whereas genetic blockage of respiration (by deleting TCA-cycle and ETC components) has the opposite effect^2^. Respiration-derived O_2_^•−^ inactivates aconitase^60,61^ with the release of free iron, which catalyzes the production of highly toxic hydroxyl radicals via Fenton chemistry^1,37,62^.

We have previously reported on the positive biochemical and physiological effects of elevated MnSod2 activity in young cells deleted for cytochrome c peroxidase (*ccp1*Δ)^48^. Like *ctt1*Δ, the *ccp1*Δ mutant exhibits low O_2_^•−^ and high H_2_O_2_ levels plus it possesses stable aconitase activity, accumulates low amounts of free iron and hydroxyl radicals, amasses mitochondrial damage more slowly and lives longer than wild-type cells^48^. These traits arise from the beneficial mitochondrial H_2_O_2_ stress response known as mitohormesis, which requires MnSod2 upregulation^17,36,37,63^. Presumably, the advantages of elevated MnSod2 activity in early log phase contribute to the increased miconazole resistance of *ctt1*Δ cells. At 24 h after miconazole treatment, the three strains possess comparable MnSod2 activity and O_2_^•−^ levels (Fig. 4). Nonetheless, based on Rhod123 staining^64^, miconazole does not increase respiration in *ctt1*Δ cells (Fig. S6), suggesting that mitohormesis protects mitochondrial function^65^.

### Catalase and azole resistance in *S. cerevisiae* vs. *C. albicans* and *C. glabrata*

Given their different catalase and Sod isozymes (Fig. 1), it is informative to compare azole sensitivity in *S. cerevisiae* and *C. albicans*. It was reported in the 1970s that fungistatic doses of miconazole induce catalase activity in *S. cerevisiae* and *C. albicans* whereas fungicidal doses inhibit this activity^14^. We confirm these results for *S. cerevisiae* but show that only Ctt1 activity is induced (Fig. 3B) since peroxisomal/mitochondrial Cta1 is repressed by glucose^66^. Cta1 is the only catalase isoform in *C. albicans*^29^ (Fig. 1), and synergistic killing of *C. albicans* biofilms by fluconazole and H_2_O_2_ has been reported but no molecular mechanism was suggested^67^.

Like *S. cerevisiae, the* opportunistic yeast *C. glabrata* possesses Cta1, cytosolic CuZnSod1 and mitochondrial MnSod2. Antifungals also induce ROS production and stimulate catalase, Sod and glutathione peroxidase activities in *C. glabrata*^21,45^. Azole resistance is associated with increased catalase activity^5^ and increased protein levels of thiol peroxidases^68^, but whether deletion of these antioxidant enzymes alters azole resistance in *C. glabrata* remains to be seen.

## Conclusions

Although high catalase activity has been linked to azole resistance in *C. albicans*, *C. glabrata* and *S. cerevisiae*, the present study reveals that azole-induced upregulation of Ctt1 activity potentiates azole toxicity by depressing MnSod2 activity in *S. cerevisiae*, Hence, MnSod2 is an interesting antifungal target in this yeast but target antioxidant enzymes are likely to be species dependent. Therefore, to expand our knowledge of the role of a given antioxidant activity in fungal survival strategies, we need to establish the potency of antifungal drugs in yeasts singly deleted for the antioxidant enzyme of interest as performed here for *S. cerevisiae*.

## Materials and Methods

### Reagents

Suppliers of chemicals/biochemical were as follows: Peptone, yeast extract, microbiological agar, phenylmethylsulfonyl (PMSF), tetramethylethylenediamine (TEMED), *tris*(hydroxymethyl)aminomethane (Tris, electrophoresis grade 99%), glycine and sodium chloride (Bioshop); glucose and 30% H_2_O_2_ (v/v) (Fisher Scientific); buffer salts, 3-amino-1,2,4-triazole (aminotriazole or ATZ), 2,2,6,6-tetramethyl-1-piperidinyloxyl (TEMPO^**•**^) and 2-(2,2,6,6-tetramethyl-1-piperidinyloxyl-4-ylamino)-2-oxoethyl triphenylphosphonium chloride (Mito-TEMPO^**•**^) (Sigma); fluconazole and miconazole nitrate (Santa Cruz Biotechnology); itraconazole, clotrimazole, posaconazole and voriconazole (Cayman Chemicals). The Bradford reagent and other electrophoresis reagents were obtained from Biorad.

### Yeast strains

The *Saccharomyces cerevisiae* wild-type and mutant BY4741 strains used in this work are listed in Table 1. The wild-type strain was purchased from the EUROSCARF. The *cta1*Δ, *ctt1*Δ, *sod1*Δ and *sod2*Δ mutant strains are derived from the Yeast Deletion Project^69,70^ and were kindly provided by Professor Christopher Brett (Department of Biology, Concordia University).

### Growth conditions and H_2_O_2_ preconditioning

Precultures (10 mL) were obtained by growing single colonies of each strain in YPD (1% yeast extract, 2% peptone and 2% dextrose) for 24 h at 30 °C with high aeration (medium-to-flask ratio of 1:5 and shaking at 225 rpm). These cultures were used to inoculate 25 mL of fresh YPD in 125-mL flasks to give the experimental cultures at an initial OD_600_ of 0.01 (OD_600_ was measured at a 1.0-cm pathlength unless otherwise indicated). Cells (3 mL) were grown under the same conditions to mid-log phase (OD_600_ 0.50; 12 h) and preconditioned with 0.2 mM H_2_O_2_ for 30 min at 30 °C/225 rpm where indicated.

### Determination of azole minimum inhibitory concentration (MIC)

The solid azoles were dissolved in 100% ethanol to give stocks of 50 mg/mL fluconazole, 10 mg/mL voriconazole, 1 mg/mL miconazole and clotrimazole; and in 100% dimethyl sulfoxide (DMSO) to give stocks of 1 mg/mL posaconazole and itraconazole. Since H_2_O_2_ preconditioning causes a 25–30% reduction in viable *ctt1*Δ cells^34^, the liquid cultures were diluted to OD_600_ 0.15 (10^6^ cfu/mL) in fresh YPD before MIC determination. Our initial cell density is higher than suggested by the Clinical and Laboratory Standards Institute (10^3^ cfu/m)^44^ to provide sufficient cells for the biochemical analyses. Cells were exposed to different azole concentrations in 96-well plates (final volume of 200 μL per well) and MICs were determined as described^44^. Briefly, cells were mixed with the drug and OD_600_ was measured on a SpectraFluor Plus Tecan plate reader at t = 0 and t = 24 h after growth at 30 °C without shaking. The MIC for each azole was determined from a plot of OD_600_ at t = 24 h minus that t = 0 vs. [azole]. The MIC is the lowest antifungal concentration that results in no detectable growth after 24 h incubation^44^. MICs for cultures simultaneously treated with the azole and 1 mM TEMPO^•^, 1 mM mito-TEMPO^•^ (Sod mimetics)^55,56^ or 25 mM aminotriazole (catalase inhibitor)^51^ were determined the same way in 96-well plates. To establish if an azole was fungicidal or fungistatic, wells containing 1xMIC and 2xMIC of the drug were serially diluted 10x after 24 h at 30 °C, plated onto YPD agar and grown for 2 days at 30 °C to measure the viable cfu/mL. A drug was considered fungicidal if 1xMIC or 2xMIC promoted a ≥10^3^-fold reduction in viable cfu^44^.

### Soluble protein extracts

Cells ±H_2_O_2_ preconditioning and ± aminotriazole exposure were diluted to OD_600_ 0.15 in 3 mL of fresh YPD ± azole in a 15-mL Falcon tube, grown at 30 °C/225 rpm for 24 h, OD_600_ values were measured, and soluble proteins were extracted as described previously^17,34^. Briefly, after centrifugation at 2000 × *g*, cells were washed 2x with 100 mM potassium phosphate buffer at pH 7.0 (KPi) containing 0.1 mM PMSF, the pellets were diluted into KPi/PMSF, and mixed with an equal volume of acid-washed glass beads (400-600 µm). Cells were disrupted by vortexing 4 × 15 s, the homogenates were spun at 13000 × *g* for 10 min at 4 °C, and the total protein concentration in the supernatants was determined by the Bradford assay with BSA as a standard^71^.

### Catalase and Sod activity assays

Cells exposed to azole concentrations below the MIC (sub-MIC) were used in the biochemical analyses to avoid the general metabolic collapse and down regulation of multiple enzyme activities seen at lethal drug concentrations^14,34^. To assay for catalase activity, 25–150 µL aliquots of soluble protein extract^72^ containing 20–100 μg protein were added to 1.0 mL of 20 mM H_2_O_2_ in 50 mM KPi in a cuvette. H_2_O_2_ decomposition was monitored at 240 nm (ε_240_ = 43.6 M^−1^ cm^−1^)^72^. One unit of catalase activity catalyzes the degradation of 1 µmol of H_2_O_2_ per min^34,72^. Sod activity was assayed using the Superoxide Dismutase Detection Kit (Cell Technologies, CSOD100), where O_2_^•−^ is generated by xanthine/xanthine oxidase and oxidized by XTT (2,3-bis(2-methoxy-4-nitro-5-sulfophenyl)-2*H*-tetrazolium-5-carboxanilide)^73^. One unit of Sod activity inhibits the rate of XTT reduction by O_2_^•−^ by 50% and was assayed according to the manufacturer’s instructions using 2–10 µg of total protein in 96-well plates. To determine MnSod2 activity only, lysates at 2 mg/mL total soluble protein were preincubated with 5 mM KCN for 30 min at room temperature to fully inhibit CuZnSod1 prior to assaying 5–50 µg of total protein for Sod activity.

### Relative ROS levels

Relative levels were estimated as we described before^17,37^ using the fluorescent probes, dihydrorhodamine 123 (DHR) for H_2_O_2_^46^ and dihydroethidine (DHE) for O_2_^•−^ ^54^. Cultures were diluted to OD_600_ 0.15 in 3 mL of fresh YPD in a 15-mL Falcon tube, vehicle (ethanol) or 0.05xMIC (0.4 μg/mL or ~1 μM) miconazole was added, cells were incubated at 30 °C/225 rpm for 8 or 24 h, harvested at 2000 × *g* for 10 min, washed once and resuspended in PBS (10 mM NaPi and 150 mM NaCl, pH 7.0) to a final density of 10^7^ cells/mL. One mL of suspension was stained with 5 µM DHR or 5 µM DHE at 30 °C for 120 and 60 min, respectively, the cells were pelleted, diluted to 10^6^ cells/mL in PBS, fixed with 2% formalin (v/v) and analyzed by flow cytometry (BD Accuri C6, BD Biosciences). The fluorescence from individual cells was measured and expressed as relative fluorescence units (RFU). Relative H_2_O_2_ and O_2_^•−^ levels are estimated from the median RFU of 10,000 cells for each sample.

### Statistical analyses

These were performed using the two-tailed Student’s t-test calculated using Graph Pad Prism 7 software. The analyses compare each sample with the wild-type untreated control (see figure legends). Probabilities < 5% are considered significant (p < 0.05).

## Supplementary information

Ctt1 catalase activity potentiates antifungal azoles in the emerging opportunistic pathogen <i>Saccharomyces cerevisiae</i>
