# Supplementary material for: Ctt1 catalase activity potentiates antifungal azoles in the emerging opportunistic pathogen Saccharomyces cerevisiae
Source: Sci Rep. 2019 Jun 24;9:9185. doi: 10.1038/s41598-019-45070-w (PMC6591360; doi:10.1038/s41598-019-45070-w)
Supplement: Supplementary file 1 — Ctt1 catalase activity potentiates antifungal azoles in the emerging opportunistic pathogen Saccharomyces cerevisiae [file 41598_2019_45070_MOESM1_ESM.pdf]

Supplemental data

Ctt1 catalase activity potentiates antifungal azoles in the emerging opportunistic pathogen *Saccharomyces cerevisiae*

Dorival Martins, Dao Nguyen and Ann M. English*

Supplementary methods

Rhodamine 123 staining: This is performed to estimate the mitochondrial membrane potential, which serves as an indicator of mitochondrial function (refs *[17, 37]* of the main text). *S. cerevisiae* (BY4741) cultures were diluted to an OD_{600} of 0.15 in 3 mL of fresh YPD, exposed to the vehicle (ethanol) or 0.05xMIC (0.4 μg/mL) miconazole at a medium-to-flask ratio of 1:5 at 30 °C/225 rpm, 24 h later cells were harvested at 2,000xg for 10 min, washed once and suspended in PBS (10 mM sodium phosphate and 150 mM NaCl, pH 7.0) to a final density of 10^7 cells/mL. One mL of suspension was stained with 5 µM Rhod123 at 30 ºC for 120 min. The stained cells were pelleted, diluted to 10^6 cells/mL in PBS, fixed with 2% formalin (v/v) and analyzed by flow cytometry (BD Accuri C6, BD Biosciences). Relative fluorescence units (RFU) of 10,000 individual cells were measured with ex/em 490/520 nm.

Table S1. Comparison of antifungal azole MICs for wild-type and *ctt1*Δ *S. cerevisiae* cells [a]

	MIC μg/mL (μM) [b]					
**Strain** [c]	***Itraconazole***	***Fluconazole***	***Posaconazole***	***Voriconazole***	***Miconazole***	***Clotrimazole***
**wild-type**	32 (45)	32 (105)	32 (46)	> 256 (> 730)	8 (19)	4 (12)
***ctt1*Δ**	64 (90)	128 (420)	256 (368)	> 256 (> 730)	64 (152)	8 (24)
ratio [d]	2	4	8	---	8	2

[a] Minimum inhibitory concentrations (MICs) for the triazoles (black font) and imidazoles (red font) were determined as outlined in the legend to Fig. 2 of the main text. Cultures were at an initial cell density of 10^6 cfu/mL when challenged with the azoles.

[b] MICs in μg/mL and μM (in parenthesis). See structures and molecular weights (MW) below.

[c] The yeast strains are described in Table 1 of the main text. [d] Ratio of MIC for *ctt1*Δ and wild-type cells.

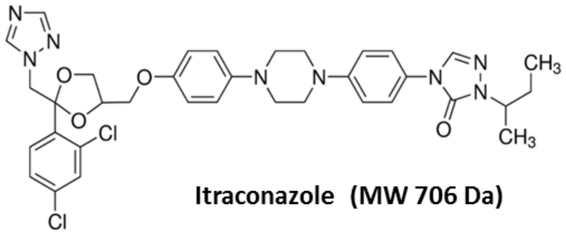

Itraconazole (MW 706 Da)

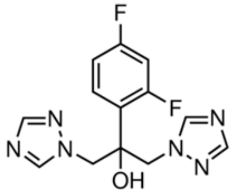

Fluconazole (MW 306 Da)

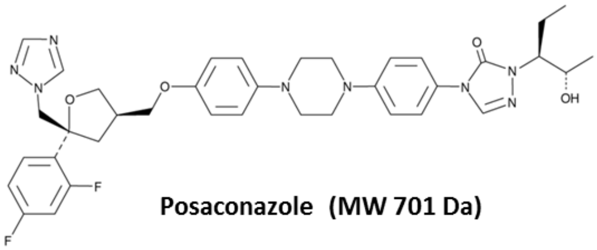

Posaconazole (MW 701 Da)

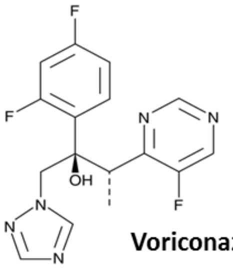

Voriconazole (MW 349 Da)

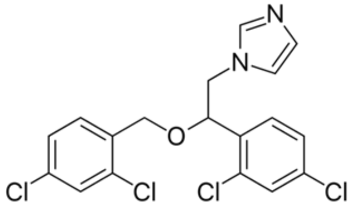

Miconazole (MW 416 Da)

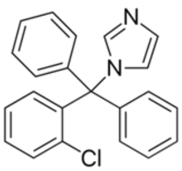

Clotrimazole (MW 345 Da)

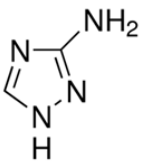

3-Amino-1,2,4-triazole (MW 84.1 Da)

Table S2. Azole classification as fungistatic or fungicidal against wild-type *S. cerevisiae* [a]

	cfu/mL					
	Itraconazole	***Fluconazole***	***Posaconazole***	***Voriconazole***	***Miconazole***	***Clotrimazole***
No azole	1.5 ± 0.3x10^6	1.4 ± 0.2x10^6	1.9 ± 0.3x10^6	NA [c]	1.4 ± 0.1x10^6	1.2 ± 0.3x10^6
1 x MIC [b]	1.6 ± 0.1x10^4	3.6 ± 1.2x10^4	1.7 ± 0.2x10^4	NA [c]	1.4 ± 0.4x10^2	4.6 ± 2.8x10^3
2x MIC [b]	1.4 ± 0.2x10^4	1.2 ± 0.3x10^4	1.7 ± 0.2x10^4	NA [c]	1.2 ± 0.3x10^1	1.6 ± 0.1x10^3
Classification [a]	Fungistatic	Fungistatic	Fungistatic	NA[c]	Fungicidal	Fungicidal

[a] An azole is classified as fungicidal under the present experimental conditions if 1xMIC or 2xMIC promotes a ≥ 10^3-fold reduction in the viable cfu/mL. Cultures containing 1xMIC and 2xMIC were serially diluted 10X after 24 h at 30 °C/225 rpm, plated onto YPD agar and grown for 2 days at 30 °C to measure the cfu/mL listed here. Note that the triazoles (black font) are fungistatic (with the exception of voriconazole) and the imidazoles (red font) are fungicidal.

[b] MICs (minimum inhibitory concentrations) were determined as outlined in the legend to Fig. 2 of the main text. Cultures were at an initial cell density of 10^6 cfu/mL when challenged with the azoles.

[c] Not applicable (NA) since at a concentration of 256 µg/mL, voriconazole has no inhibitory effect on the growth of wild-type cells (see Table S1).

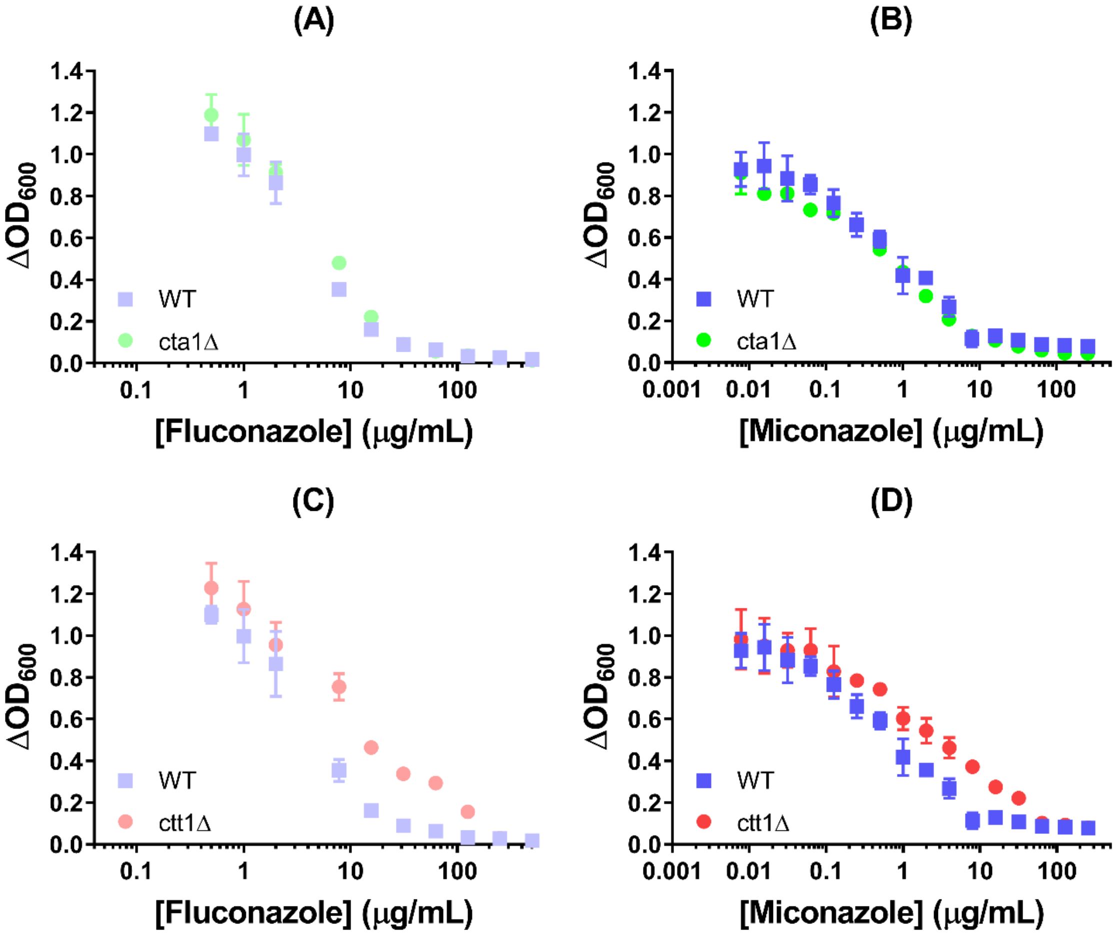

Fig. S1. *Minimum inhibitory concentration (MIC) of fluconazole and miconazole for wild-type, cta1Δ and ctt1Δ S. cerevisiae cells.* OD_{600} values were measured in 96-well plates at t= 0 and t= 24 h after azole addition to the cultures, and panels compare the ΔOD_{600} values (24 – 0 h) of wild-type ■ to (A, B) *cta1Δ* ● and (C, D) *ctt1Δ* cells ● exposed to increasing concentrations of fluconazole and miconazole (see Fig. 2 of the main text). Table 2 of the main text lists the MIC for each sample. The results represent the avg ± SD of six independent replicates (n = 6). Representative values determined in the plate reader are shown in Fig. S7.

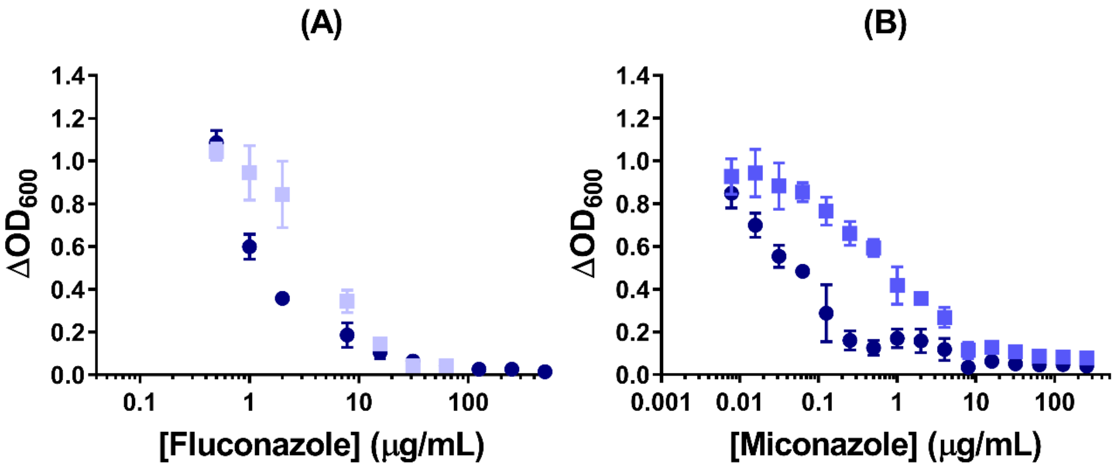

Fig. S2. *H_2O_2 preconditioning decreases the fluconazole and miconazole MIC of wild-type S. cerevisiae cells.* Cells were grown to an OD_{600} of 0.50 (12 h) at 30 °C/225 rpm at a media-to-flask ration of 1:5, then 0.2 mM H_2O_2 ● or 0.85% aqueous NaCl ■ (control) was added, after 30 min at 30 °C/225 rpm the cells were diluted to an OD_{600} of 0.15 and exposed to increasing concentrations of (A) fluconazole or (B) miconazole in 96-well plates. MICs were determined (see caption to Fig. S1) and the values are summarized in Table 2 of the main text. The results represent the avg ± SD of six independent replicates experiments (*n* = 6).

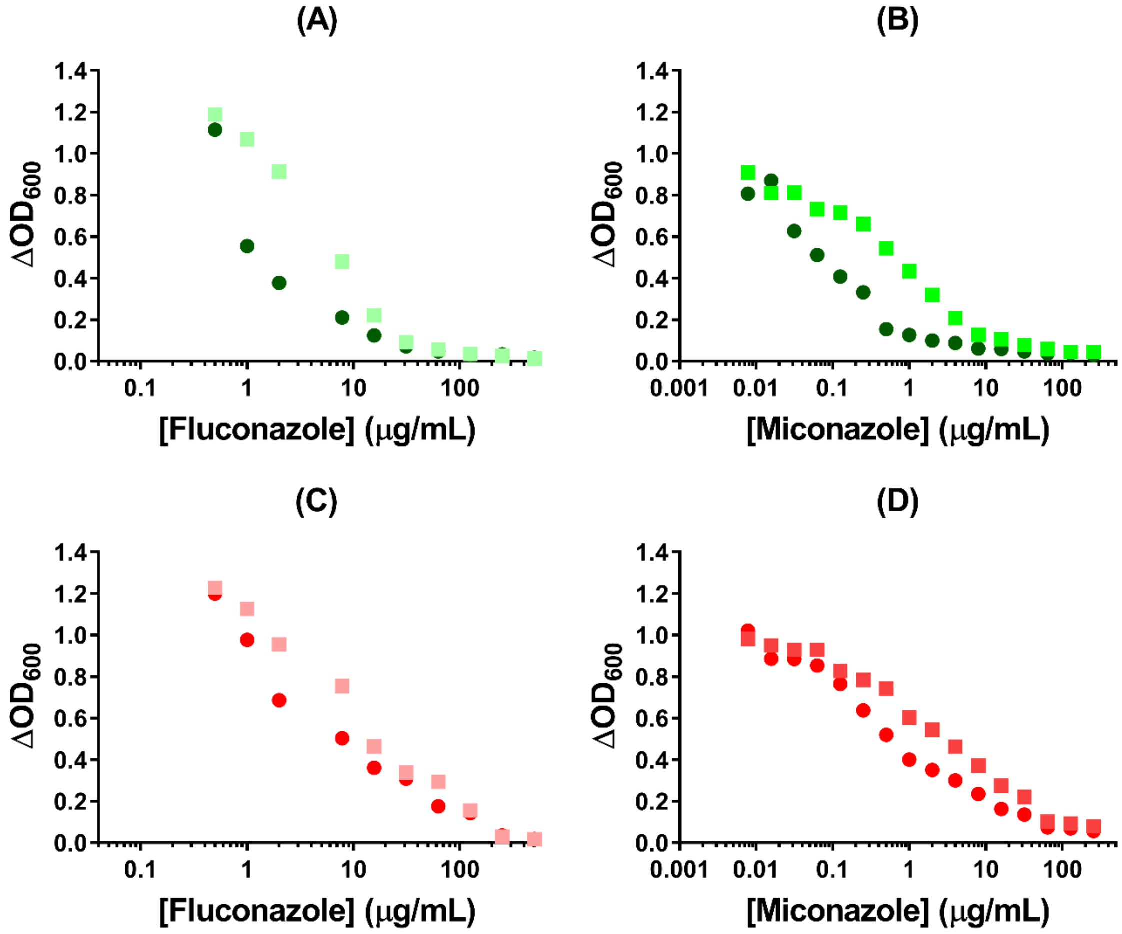

Fig. S3. *H_2O_2-preconditioning decreases the fluconazole and miconazole MIC of cta1Δ cells but does not alter that of ctt1Δ cells.* Cells (*S. cerevisiae*) were grown and preconditioned with 0.2 mM H_2O_2 ● or 0.85% aqueous NaCl ■ (control) as described in the caption to Fig. S2. After 30 min (A, B) *cta1*Δ ■● and (C, D) *ctt1*Δ ■● cells were diluted into fresh YPD to an OD_{600} of 0.15 and treated with fluconazole and miconazole in 96-well plates. MICs were determined (see caption to Fig. S1) and the values are summarized in Table 2 of the main text. The results represent the avg ± SD of six independent experiments (*n* = 6).

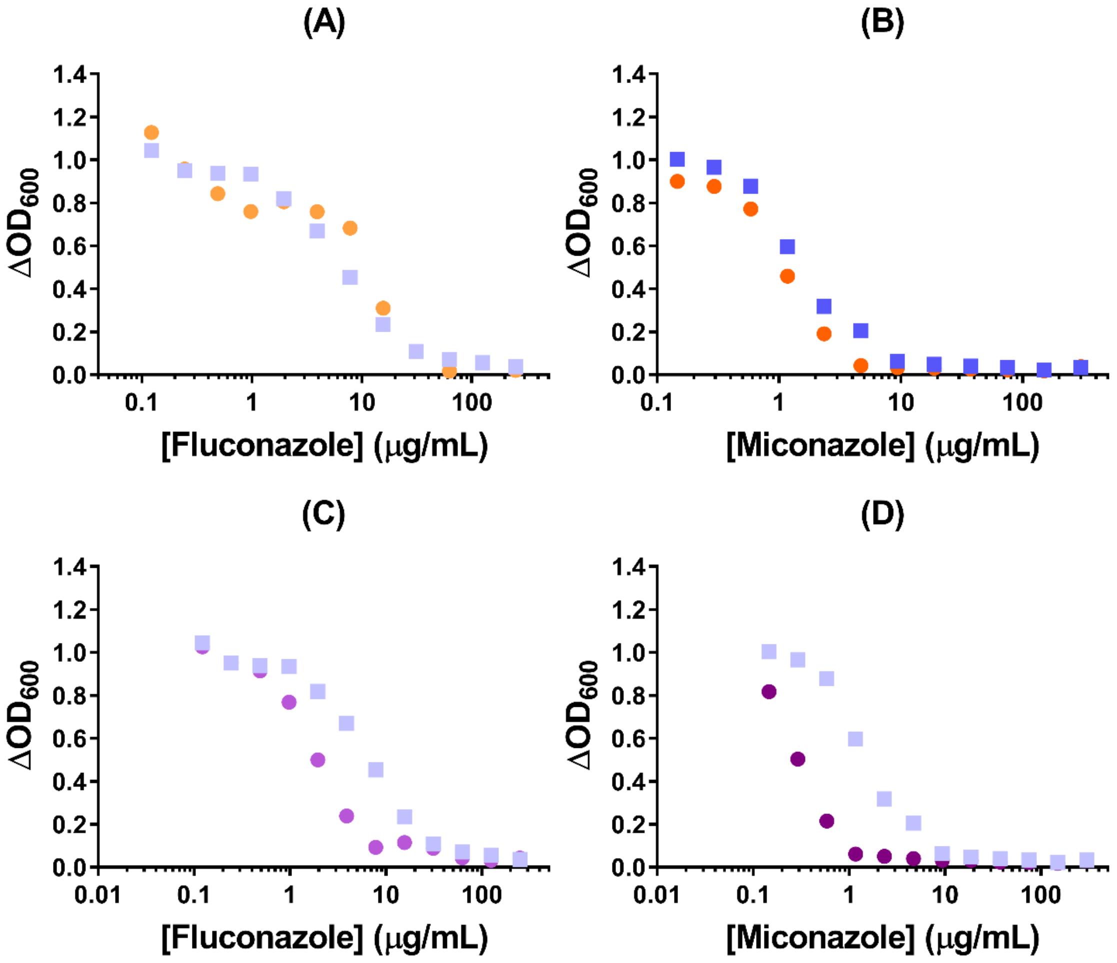

Fig. S4. *Deletion of MnSod2, but not deletion of CuZnSod1, decreases the fluconazole and miconazole MIC of S. cerevisiae cells.* Wild-type ■ and (A, B) *sod1*Δ ● or (C, D) *sod2*Δ ● cells were exposed to increasing concentrations of (A, C) fluconazole (B, D) miconazole in 96-well plates. MICs were determined (see caption to Fig. S1) and the values are summarized in Table 2 of the main text. The results represent the avg ± SD of six independent experiments (*n* = 6).

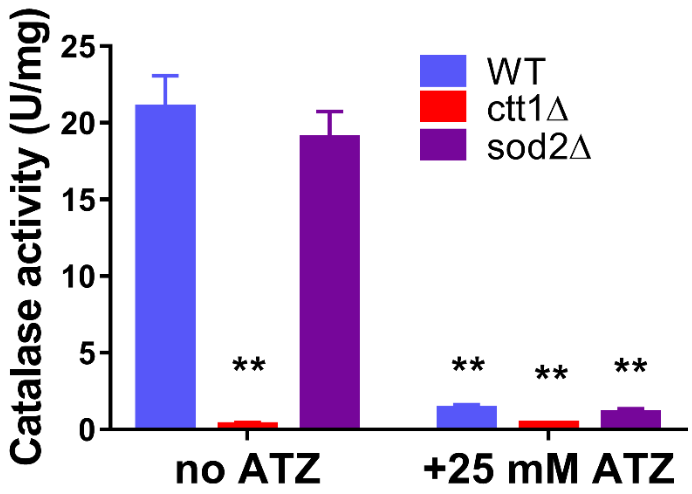

Fig. S5. *Aminotriazole (ATZ) inhibits catalase activity in wild-type and sod2Δ S. cerevisiae cells.* Wild-type ■, *ctt1*Δ ■ and *sod2*Δ ■ cells grown to OD_{600} 0.50 (12 h) in YPD at a medium-to-flask ratio of 1:5 at 30 °C/225 rpm were diluted to OD_{600} 0.15 (10^6 cfu/mL) and incubated with 25 mM aminotriazole (ATZ) for 24 h at 30 °C/225 rpm. Extraction of soluble protein and catalase activity were performed as described under *Materials and methods* of the main text. Results are the avg ± SD of three independent experiments (n = 3). Statistical analyses performed using Student's t-test compare each sample with the wild-type untreated control (no ATZ). * $p < 0.05$ and ** $p < 0.01$.

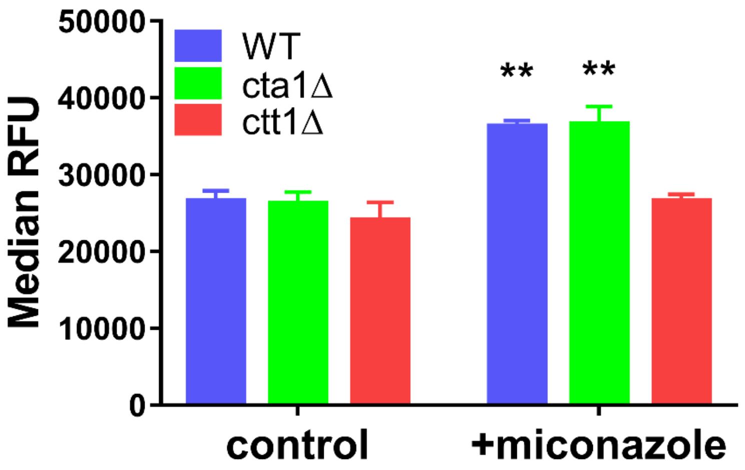

Fig. S6. *Miconazole increases the mitochondrial membrane potential in wild-type and cta1Δ but not in ctt1Δ S. cerevisiae cells.* Cells at initial OD_{600}= 0.15 were grown ± 0.05xMIC (0.4 μg/mL) miconazole in YPD at 30 °C/225 rpm. After 24 h, the cells were stained with Rhod123 and the relative fluorescence units (RFU) of 10,000 individual cells per sample were measured with ex/em 490/520 using flow cytometry as outlined in the *Supplementary methods.* The median RFU estimates a sample's relative Rhod123 accumulation. Results represent the avg ± SEM of six independent experiments (n = 6). Statistical analyses performed using Student's t-test compare the wild-type control (ethanol vehicle only) vs. miconazole treated samples * $p < 0.05$ and ** $p < 0.01$. Note that an increase in the medium RFU signifies an increased Rhod123 accumulation due to an increase in mitochondrial membrane potential. Therefore, miconazole increases the membrane potential in wild-type and *cta1*Δ cells, but not *ctt1*Δ cells, revealing increased mitochondrial function and increased respiration (ref [64] of the main text).
